# Patent Medicines

**Published:** 1906-06-09

**Authors:** 


### Patent Medicines.

A most praiseworthy campaign is at present being waged in the American Press against perhaps one of the greatest frauds of modern times—namely, patent medicines. Perhaps the facts thus brought to light do not apply in their entirety to the patent-medicine trade in this country, but so many of them do that we think it high time that the attention of our readers should be drawn to the enormous traffic in patent medicines in this country and to the dangers both to the public health and to the public morality likely to accrue so long as the present state of affairs continues. The facts are amazing. In America £20,000,000 annually is spent by the population upon patent medicines, and of this sum it is computed that £8,000,000 goes to the advertising departments of the newspapers. At the present time in Great Britain there are no less than 40,000 makers or vendors of patent medicines, and these patent medicines are computed to supply a revenue of £331,000 to the State.

The patent medicine dealer reaps his harvest from so-called incurable disease. Consumption cures, cancer cures, epilepsy cures, paralysis cures, make up the large majority of patent medicines. If we add to these headache cures, tonics, laxatives, and pick-me-ups, we have practically completed the list. Practically all the headache cures contain acetanilid—a very powerful drug, the maximum dose of which in the pharmacopœia is 3 grains, but there are numerous headache cures on the market containing in each dose an amount greatly in excess of this. A dose, for instance, of bromo-seltzer is estimated to contain about 10 grains. Some analyses give the acetanilid content of anti-camnia as high as 68 per cent. It is a known fact that even when this drug is prescribed by experienced physicians cardiac failure and acute blood changes not infrequently happen; what then must be said of the effect upon the community of the indiscriminate use of nostra containing exalted doses of this active poison?

Cocaine is a very frequent constituent of patent medicines, being used often for its anæsthetic and astringent properties. The public soon, however, find out that the patent medicines which contain cocaine have a markedly stimulant and exhilarating effect. From indirect evidence it appears certain that the cocaine-containing nostra find their most extensive clientèle among women. Some of these preparations, which are generally vaunted as curing catarrhs, contain as much as 4 per cent. of cocaine. The subject of the cocaine habit is unfortunately well known to the practising physician, and a more miserably degraded human being it is difficult to find or even imagine. How frequently the uneradicable germs of the cocaine habit have been planted and nourished by the use of the cocaine contained in patent medicine we shall probably never know, but that the youth of this country should require no more elaborate key to an inexhaustible store of this insidious and subtle exhilarant than the possession of a few shillings should surely be a matter at once of horror and concern.

The third constituent of patent medicines is alcohol. The sole active constituent of many of these preparations is alcohol, flavoured and perhaps disguised, but still therapeutically and gastronomically alcohol. Some patent medicines contain as much as 41 per cent. of alcohol, or are approximately as strong as brandy. These remedies never mention alcohol in their title, and by means of them a staunch teetotaler may purchase a feeling of bien-être with a strong dose of alcohol. Indeed, one often finds testimonials praising these alcoholic patent medicines, written in ignorance by staunch supporters of total abstinence. It is scarcely needful to say that the above is a very popular class of patent medicines.

The last important constituent of these remedies which we shall notice is opium and its derivatives. This drug, along with prussic acid and chloroform, figures very largely as the active principle of all cough mixtures, anti-diarrhœa potions, and soothing syrups. Sometimes a very considerable quantity of this substance and its alkaloidal derivatives is contained in these syrups, and quite frequently deaths from typical opium-poisoning have been reported in children from their use, as has also the development of the opium or morphine habit in adults. Not infrequently chloral is associated with the above narcotics and produces its own peculiar raids upon the morale of the habitual consumer.

Our American contemporary makes an eloquent plea for legislation to prohibit this deplorable traffic. Such legislation must, in the first instance, prohibit the sale without a physician's prescription of any but the smallest doses of active drugs, and must demand full declaration on the label of the contents of every such preparation.

